# Cognitive heterogeneity, health dynamics, and subjective well-being across adulthood: evidence from the German SOEP longitudinal panel

**DOI:** 10.1186/s12889-026-29407-5

**Published:** 2026-09-11

**Authors:** Kerstin Erdal, Johanna Finnemann

**Affiliations:** 1 Department of Psychiatry, Institute of Neuroscience and Physiology, Sahlgrenska Academy of University of Gothenburg, Medicinaregatan 3, Gothenburg, Västra Götaland 413 90 Sweden; 2 Department of Work & Organizational Psychology, Institute for Psychology, University of Hagen, Hagen, Germany

**Keywords:** Self-rated health, Life satisfaction, Cognitive profiles, German Socio-Economic Panel (SOEP), Longitudinal analysis

## Abstract

**Background:**

Self-rated health is an important correlate of life satisfaction, but the strength of this association varies across individuals. The present study examined whether cognitive clusters derived from performance-based measures of verbal knowledge and response-time characteristics moderate the within-person association between changes in self-rated health and life satisfaction.

**Methods:**

Performance-based cognitive measures from the German Socio-Economic Panel Innovation Sample (SOEP-IS) were used to derive cognitive profiles based on verbal knowledge, processing speed, and processing-speed variability. Cognitive profiles assessed in 2014 were linked prospectively to longitudinal observations from the German Socio-Economic Panel (SOEP-CORE) collected between 2014 and 2023. Random-effects within-between linear mixed-effects models examined the association between within-person changes in self-rated health and life satisfaction and tested whether this association was moderated by cognitive profile. Additional between-person analyses examined associations between cognitive profiles and self-rated health, life satisfaction, and affect.

**Results:**

Cognitive clusters were associated with between-person differences in self-rated health and subjective well-being. In the primary longitudinal model, within-person improvements in self-rated health were associated with small increases in life satisfaction, although this association was attenuated in the survey-weighted sensitivity analysis. Cognitive clusters did not explain individual differences in how changes in self-rated health translated into changes in life satisfaction over time, a finding that was consistent across sensitivity analyses.

**Conclusions:**

Findings suggest that the cognitive performance measures examined here are associated with between-person differences in self-rated health and subjective well-being but do not systematically account for individual differences in how life satisfaction responds to changes in self-rated health over time.

**Supplementary Information:**

The online version contains supplementary material available at https://doi.org/10.1186/s12889-026-29407-5.

## Introduction

Self-rated health (SRH) captures individuals’ global assessment of their physical and mental health and integrates information about functioning, symptoms, and well-being. Across population-based studies, SRH is a strong predictor of mortality, health care utilization, and functional decline, often performing comparably to more detailed objective indicators [1, 2]. In longitudinal research, SRH is also sensitive to within-person change, making it a useful indicator for studying how individuals experience health improvements and declines over time [3, 4].

Rather than reflecting a single health dimension, SRH has been conceptualized as a multidimensional evaluative process in which respondents integrate information about symptoms, functional limitations, chronic conditions, bodily sensations, and psychological well-being into an overall judgment of their health [5]. Producing this appraisal requires cognitive processing, and individuals experiencing higher cognitive load can experience greater difficulty evaluating and reporting their health accurately [6, 7].

Life satisfaction represents a cognitive, evaluative judgment of one’s overall quality of life [8]. Although health is a key correlate of life satisfaction, longitudinal evidence suggests that individuals differ substantially in how changes in self-rated health translate into changes in evaluative well-being [9]. Some individuals show pronounced changes in life satisfaction in response to changes in self-assessed health, whereas others exhibit relative stability despite life events such as disability [10, 11]. Vulnerability–stress models provide one framework for understanding such individual differences, proposing that relatively stable individual characteristics can shape responses to changing circumstances or stressors [12, 13]. From this perspective, the same change in health may have different consequences for well-being depending on the resources or vulnerabilities an individual brings to that experience. One potential source of such heterogeneity is cognitive functioning.

Cognitive abilities are relatively stable across early and mid-adulthood, with gradual age-related changes that are often compensated for by accumulated knowledge and experience [14, 15]. In population-based research, higher cognitive functioning has been associated with better health, higher quality of life, and lower risk of functional impairment [16, 17]. Additionally, a substantial body of longitudinal research has examined cognitive functioning in later life, including healthy cognitive ageing, cognitive reserve, cognitive decline, and associations between cognition, health, and quality of life (e.g., [18–20]).

Higher performance on verbal knowledge tasks has been associated with greater cognitive reserve, with vocabulary knowledge often used as a marker of cognitive reserve in epidemiological and aging research [21, 22]. Cognitive reserve broadly refers to individual differences in the capacity to maintain cognitive functioning in the presence of age- or disease-related brain changes, potentially through flexible and efficient use of cognitive resources [23, 24]. From this perspective, cognitive abilities may provide resources that support adaptation when individuals encounter changing health-related demands.

Processing speed may be particularly relevant to adaptation because it represents a fundamental dimension of cognitive aging and is associated with performance across multiple cognitive domains and functional independence in later life [25–27]. Processing speed has also been proposed as one potential mechanism underlying associations between general cognitive ability and mortality [28, 29]. According to processing-speed theory, slower processing can constrain higher-order cognition by limiting the amount of information that can be processed and maintained during cognitive operations [26]. Processing efficiency may therefore influence how effectively individuals evaluate and respond to changing demands.

Such cognitive resources may be relevant when adapting to changes in health. The dual-process model of coping distinguishes between modifying circumstances in pursuit of valued goals (assimilation) and adjusting goals and standards when circumstances cannot readily be changed (accommodation; [30]). Verbal knowledge may facilitate comprehension of health information and communication about health, whereas processing speed may support the efficient evaluation of and response to changing health-related demands. Consistent with a potential role for cognitive abilities in well-being, verbal working memory and processing speed have been associated with subsequent changes in life satisfaction [31, 32], although evidence is not uniform [33]. Direct evidence linking specific cognitive perfomance to adaptive coping processes nevertheless remains limited.

Taken together, these perspectives suggest that different cognitive abilities may provide partly distinct resources for adapting to health-related challenges. Individuals with similar levels of overall cognitive functioning may therefore differ in their capacity to comprehend, evaluate, and respond to changes in health. Such differences could, in turn, influence how strongly changes in perceived health are reflected in life satisfaction. Accordingly, we expected individuals with greater verbal knowledge and more efficient processing to show reduced sensitivity of life satisfaction to health declines, as greater cognitive resources may facilitate coping, compensation, and the appraisal of changing health conditions.

Previous research has primarily examined associations between cognitive performance and health or subjective well-being, typically focusing on between-person differences in overall cognitive functioning. Far less attention has been given to whether independently measured, task-based cognitive performance moderates within-person associations between changes in self-rated health and life satisfaction. The present study addresses this gap using longitudinal data from the German Socio-Economic Panel (SOEP; [34]) linked with performance-based cognitive measures from the SOEP Innovation Sample (SOEP-IS, [35]). Cognitive profiles were derived from assessments of verbal knowledge, mean response latency, and response-time variability administered in 2014 and treated as time-invariant indicators from the year of assessment onward. To avoid retrospectively assigning these profiles to earlier outcomes, all longitudinal analyses were restricted to person-year observations from 2014 onward.

Specifically, we addressed two research questions:

- RQ1. Are within-person changes in self-rated health associated with changes in life satisfaction?
- RQ2. Do cognitive profiles moderate the within-person association between changes in self-rated health and life satisfaction?

In addition to these primary longitudinal analyses, we conducted exploratory between-person analyses examining associations between cognitive profiles and self-rated health, life satisfaction, and affect.

## Methods

### Data sources

We combined data from the German Socio-Economic Panel (SOEP-CORE) and the SOEP Innovation Sample (SOEP-IS; [35]). SOEP-CORE is a nationally representative longitudinal household survey of the population living in Germany that has collected annual information on health, well-being, and socioeconomic conditions since 1984 [34, 36]. Cognitive performance measures were obtained from the SOEP-IS cognition module administered in 2014, while longitudinal outcomes and covariates were drawn from annual SOEP person-level data. Panel information was obtained from the individual questionnaire file (*pl*) and generated person file (*pgen*), and demographic characteristics and survey weights were obtained from the biographical file (*ppathl*). Datasets were linked using the unique person identifier (*pid*).

### Sample construction

**Fig. 1:**
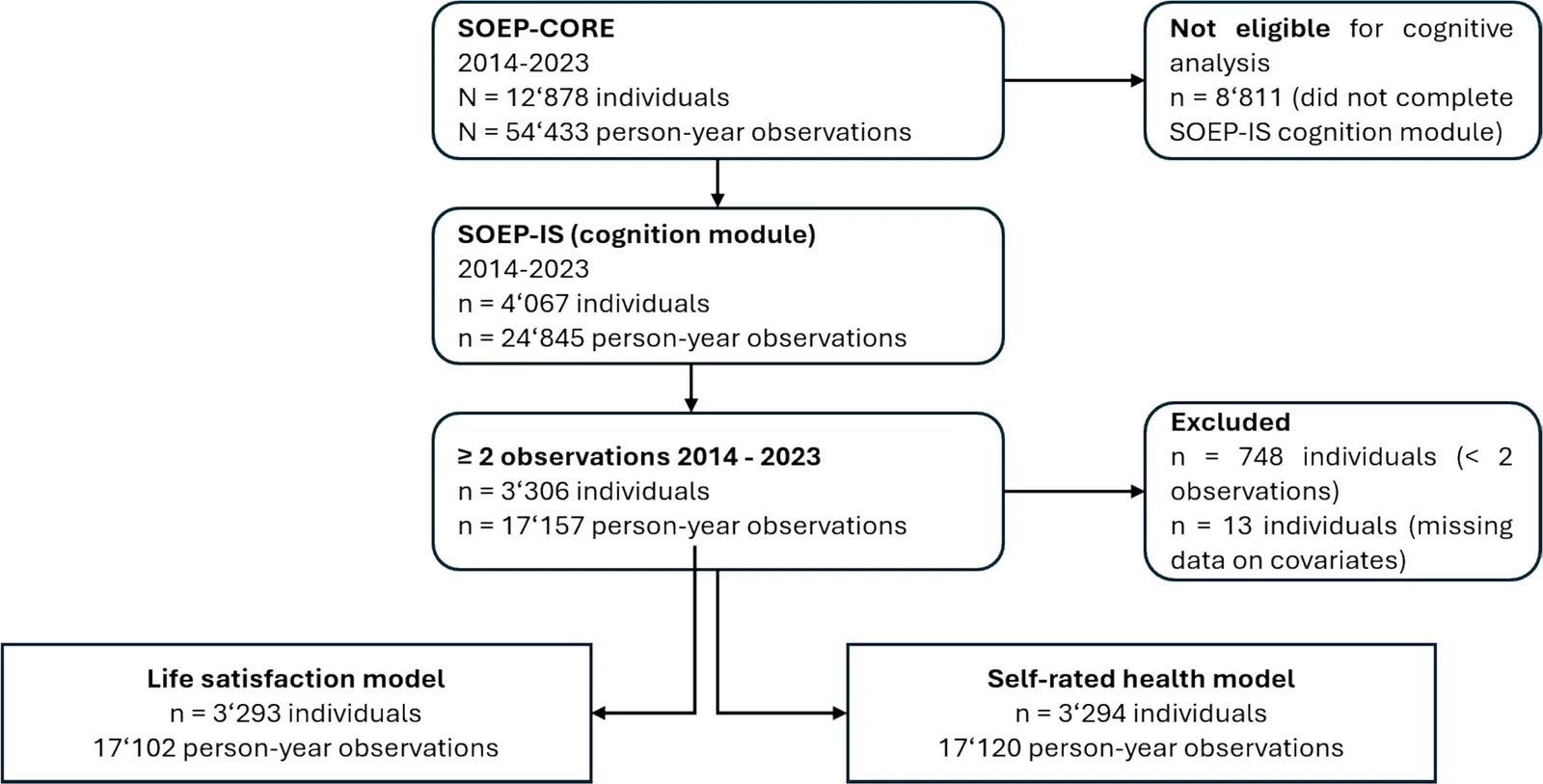
Flow diagram of participant selection for the longitudinal analyses *Note.* Cognitive profiles were derived from respondents completing the 2014 SOEP-IS cognition module. Longitudinal analyses were restricted to observations collected from 2014 onward. Respondents were required to contribute at least two model-eligible observations. Final analytic samples additionally satisfied complete-case requirements for all model variables

Cognitive profiles were derived from 4,067 respondents who completed the SOEP-IS cognition module in 2014 and were linked to the longitudinal SOEP person-level data using *pid*. To avoid retrospectively assigning cognitive profiles to outcomes measured before the cognitive assessment, all longitudinal analyses were restricted to observations from 2014 onward before constructing lagged self-rated health and the within-between decomposition (see Fig. 1 for flow chart). Analyses used complete-case estimation, with all nested models estimated on a common analytic sample. Cognitive profiles were therefore treated as time-invariant baseline indicators only during the subsequent observation period.

For the longitudinal analyses, respondents were required to contribute at least two model-eligible observations and to have complete data on all model variables. The final life-satisfaction model included 3,293 individuals contributing 17,102 person-year observations between 2015 and 2023. The corresponding self-rated-health model included 3,294 individuals contributing 17,120 person-year observations. Comparison of respondents included in and excluded from the final life-satisfaction analysis indicated some evidence of selective retention. Included respondents were somewhat older (SMD = 0.19) and had higher word-test scores (SMD = 0.27). Women were more prevalent among included than excluded respondents (53.1% vs. 47.0%, *p* = 0.003), and the overall distribution of cognitive profiles differed between groups, χ^2^(2) = 32.04, *p* < 0.001, with Profile 1 less prevalent among included respondents (16.9% vs. 25.6%). Other baseline differences were small and not statistically significant (Supplementary Table S4).

The final life-satisfaction analyses (RQ1 and RQ2) included 3,293 respondents contributing 17,102 person-year observations. Negative SOEP missing and filter codes were recoded as missing before variable construction, and no missing values were imputed. Detailed information on variable-level missingness and comparisons between respondents included in and excluded from the final analytic sample is provided in Tables S2–S4.

### Measures

#### Cognitive functioning

Cognitive functioning was assessed using two performance-based tasks from the SOEP-IS cognition module administered in 2014.

Verbal knowledge was measured using the SOEP-IS vocabulary test (f025r), in which respondents were asked to identify the single real German word among four pseudo-words. Processing speed was operationalized using item-level response times from the SOEP-IS symbol–digit task (f099time) and summarized as the respondent-specific mean reaction time across valid items; reaction times were log-transformed to reduce right skew, with higher values indicating slower responding. Although task instructions emphasized accuracy rather than speed, response times were used as an index of relative response latency under low time pressure rather than maximal speed of performance. Processing speed variability was captured as the within-task standard deviation of reaction times, reflecting response consistency. All cognitive indicators were z-standardized prior to clustering. Detailed information on task design, instructions, and coding is provided in the SOEP-IS Survey Paper by the SOEP-IS Group [35].

#### Cognitive profiles

Cognitive profiles were identified using *k*-means clustering applied to standardized verbal knowledge, log-transformed mean response latency, and response-time variability. Clustering was performed with multiple random starts and a fixed random seed to reduce sensitivity to initialization and ensure reproducibility. The number of clusters was selected based on substantive interpretability and inspection of the within-cluster sum of squares (elbow criterion, see Appendix S1). A three-cluster solution yielded well-separated and substantively interpretable profiles and was retained for analysis. A profile-based approach was used to capture distinct dimensions of performance rather than assuming levels of a single underlying cognitive ability. The resulting profiles therefore represent different configurations of verbal knowledge, mean response latency, and response-time variability rather than an ordinal continuum from low to high cognitive functioning. Cluster membership was stored as a categorical baseline indicator and carried forward across subsequent panel waves.

#### Life satisfaction

Life satisfaction was measured annually using the SOEP CORE item *plh0182*, assessed on an 11-point scale ranging from 0 (“completely dissatisfied”) to 10 (“completely satisfied”). Values outside the valid range were treated as missing. Life satisfaction was the primary outcome in the longitudinal panel analyses.

#### Self-rated health

Self-rated health was measured using the SOEP CORE item *ple0008* on a five-point scale (1 = very good to 5 = very bad). For interpretability, the item was reverse-coded so that higher values indicate better health. To distinguish within-person health changes from stable between-person differences, a within–between (random-effects within-between; REWB) decomposition of lagged self-rated health was applied. This yielded a between-person component (the individual-specific mean of lagged health) and a within-person component (the deviation from that mean).

#### Affect

Affective well-being was assessed by asking respondents how often they had experienced specific feelings during the previous four weeks, using a five-point scale ranging from 1 (“very rarely”) to 5 (“very often”). Negative affect was calculated as the mean of feeling angry (*plh0184*), worried/anxious (*plh0185*), and sad (*plh0187*), requiring at least two valid items. Positive affect was represented by the single item assessing feeling happy (*plh0186*). Affect was examined as an outcome in the exploratory between-person analyses and was additionally included as an adjustment variable in supplementary longitudinal analyses.

#### Covariates

All longitudinal models adjusted for educational attainment, wave-specific centered age, sex, and survey-year fixed effects. Educational attainment was included because of its associations with cognitive performance, health, and subjective well-being [37, 38]. Age and sex were included because both are associated with self-rated health and subjective well-being [39–42]. Survey-year fixed effects accounted for secular trends common to all respondents.

### Statistical analysis

#### Longitudinal panel models

Life satisfaction was analyzed using linear mixed-effects models with repeated observations nested within individuals. All models included random intercepts at the individual level. In the main specifications, random slopes for the within-person health component were included to allow individuals to differ in their sensitivity of life satisfaction to health changes. Models were estimated using maximum likelihood. The temporal restriction was imposed before constructing the lagged-health and within–between variables. Thus, the earliest health observation that could contribute to the lagged predictor was measured in 2014, and the earliest outcome observations entering the lagged models occurred in 2015. Models therefore estimate prospective associations during the period following the cognitive assessment.

#### Cognitive profile moderation

To examine whether cognitive functioning moderated responses to health changes, models included an interaction between the within-person health deviation and cognitive profile membership. Cluster 2 (for performance characteristics, see Table 1) served as the reference category. Moderation was evaluated from the interaction terms between within-person health and cognitive profile membership. Random-intercept and random-slope specifications were estimated on the same analytic sample, and their fit was compared using a likelihood-ratio test.

**Table 1:** Cognitive performance characteristics of the three cognitive profiles (SOEP-IS)

Cluster profile	*n*	Verbal-knowledge test score M (SD)	Processing speed M (SD)	Speed variability M (SD)
Cluster 1	756	23.60 (3.69)	3.88 (0.07)	26.40 (1.98)
Cluster 2 (ref.)	2,289	31.20 (1.88)	3.86 (0.06)	26.50 (1.77)
Cluster 3	1,022	30.90 (2.30)	3.98 (0.06)	25.10 (1.93)

*Note.* Verbal knowledge performance was measured using the SOEP-IS vocabulary test. Processing speed represents the log-transformed mean reaction time across symbol–digit items, where higher values indicate slower processing. Speed variability reflects the within-task standard deviation of reaction times. All indicators were z-standardized prior to clustering. Cluster membership was defined using k-means clustering (*k* = 3, 50 random starts) based on the 2014 SOEP-IS cognition module

To assess the robustness of the profile-based approach, supplementary latent profile models with two to six requested profiles were estimated using the same cognitive indicators (Supplementary Table S8). The analyses did not identify an unambiguously superior alternative classification. Among solutions in which all requested profiles were occupied, BIC improved from the two- to the four-profile solution, although the smallest profile in the four-profile solution comprised only 2.5% of the sample. Higher-order solutions were degenerate, yielding fewer occupied profiles than specified. Although the requested six-profile solution yielded a numerically higher BIC, only five profiles were occupied and the smallest comprised just 0.1% of the sample. Accordingly, the latent profile analyses were treated as a sensitivity assessment rather than as independent validation of the three-cluster solution. The three-cluster *k*-means solution was retained as a parsimonious and substantively interpretable representation of the cognitive indicators.

#### Simple slopes and sensitivity analyses

Cluster-specific within-person health slopes were estimated using marginal trends, with pairwise contrasts computed using Bonferroni-adjusted *p*-values. Because testing variance components involves parameters on the boundary of the parameter space, likelihood-ratio tests were supplemented with profile-likelihood confidence intervals for the random-slope standard deviation. Two sensitivity analyses were conducted. First, the primary life-satisfaction model was re-estimated using SOEP cross-sectional person weights (*phrf*) applied as prior weights at the person-year level; weights were standardized to a mean of one and trimmed at the 1 st and 99th percentiles to improve numerical stability. Second, the primary life-satisfaction model was additionally adjusted for positive and negative affect.

### Software

All analyses were conducted in R version 2025.01, using the packages *dplyr*, *tidyr*, *lme4*, and *emmeans*.

### Ethics approval

This study is based on secondary analysis of data from the German Socio-Economic Panel (SOEP), collected by the German Institute for Economic Research (DIW Berlin). The SOEP data are fully anonymized and were collected with informed consent from all participants in accordance with the ethical standards and legal requirements applicable in Germany. The authors accessed the data under a formal data use agreement with DIW Berlin. As the study involved only secondary analysis of anonymized data, no approval from an institutional ethics committee was required.

## Results

### Sample characteristics and cognitive profiles

The longitudinal analyses were based on SOEP-CORE panel data merged with cognitive profiles derived from the SOEP-IS assessment.

Cognitive profile membership was treated as time-invariant and defined based on the SOEP-IS cognition module. Of respondents who had completed the COGNIT module of the SOEP-IS sample, 56.3% belonged to the reference profile (Cluster 2), 18.6% to Cluster 1, and 25.1% to Cluster 3. Table 1 presents descriptive characteristics of the three cognitive profiles identified in the SOEP-IS sample (*N* = 4,067). Cluster 2 exhibited the highest verbal-knowledge scores and the fastest average response times. Cluster 1 was characterized by lower verbal-knowledge scores and approximately average response-time characteristics. Cluster 3 showed similarly high verbal-knowledge scores but slower average response times and lower response-time variability.

Table 2 presents descriptive statistics for life satisfaction, self-rated health, and affect across the three clusters. Participants in Cluster 1 reported slightly lower life satisfaction and self-rated health and higher negative affect than participants in the reference cluster, whereas differences between Clusters 2 and 3 were comparatively small.

**Table 2:** Descriptive statistics for self-rated health and subjective well-being by cognitive cluster

Cluster	n	Life satisfaction, *M (SD)*	Self-rated health, *M (SD)*	Negative affect, *M (SD)*	Positive affect, *M (SD)*
Cluster 1	756	7.31 (1.79)	3.38 (0.98)	2.34 (0.80)	3.56 (0.89)
Cluster 2 (reference)	2,289	7.52 (1.57)	3.50 (0.94)	2.24 (0.74)	3.68 (0.80)
Cluster 3	1,022	7.44 (1.71)	3.28 (0.95)	2.16 (0.76)	3.54 (0.84)

*Note.* Descriptive statistics are based on the 2014 SOEP-IS cognition sample. Higher self-rated health scores indicate better health. Cluster 1 was characterized by lower verbal knowledge and approximately average response-time characteristics; Cluster 2 by higher verbal knowledge and faster response times; and Cluster 3 by higher verbal knowledge, slower response times, and lower response-time variability

### Within-person health changes and life satisfaction (RQ1)

To address RQ1, we examined whether within-person changes in self-rated health were associated with changes in life satisfaction. Random-intercept and random-slope within–between (REWB) models were estimated using lagged self-rated health.

In the random-intercept model, better-than-usual health at the previous observed wave was associated with higher subsequent life satisfaction. In the random-slope model, better-than-usual self-rated health was associated with slightly higher subsequent life satisfaction (*B* = 0.054, *SE* = 0.022, 95% CI [0.011, 0.096], *p* = 0.014). At the between-person level, individuals with better average self-rated health reported substantially higher life satisfaction (*B* = 0.988, *SE* = 0.026, 95% CI [0.936, 1.040], *p* < 0.001).

Allowing the within-person health effect to vary across individuals improved model fit compared with the random-intercept model (Δχ^2^(2) = 25.54, *p* < 0.001). The estimated random-slope variance was 0.061 (*SD* = 0.246), with a 95% profile-likelihood confidence interval for the random-slope standard deviation of [0.180, 0.304]. Together, these results indicate inter-individual heterogeneity in the within-person association between self-rated health and life satisfaction.

### Cognitive profiles as moderators of the health–life satisfaction association (RQ2)

To address RQ2, we examined whether cognitive profiles moderated the within-person association between self-rated health and life satisfaction by including interactions between the within-person health component and cognitive profile membership. Compared with the reference profile (Cluster 2), neither interaction was statistically significant (Cluster 1: *B* = − 0.039, *SE* = 0.045, 95% CI [− 0.127, 0.049], *p* = 0.386; Cluster 3: *B* = − 0.007, *SE* = 0.040, 95% CI [− 0.085, 0.072], *p* = 0.869; Table 3).

**Table 3:** Random-effects within-between (REWB) models predicting life satisfaction

Panel A. Fixed effects						
**Variable**	**Random intercept model *B* (SE)**	**95% CI**	***p***	**Random slope model *B* (SE)**	**95% CI**	***p***
Intercept	4.189 (0.101)	[3.991, 4.387]	<.001	4.197 (0.101)	[3.999, 4.395]	<.001
Within-person health (lagged)	0.057 (0.021)	[0.017, 0.098]	.005	0.054 (0.022)	[0.011, 0.096]	.014
Between-person health (mean)	0.990 (0.026)	[0.938, 1.042]	<.001	0.988 (0.026)	[0.936, 1.040]	<.001
Cluster 1 (vs. Cluster 2)	–0.049 (0.054)	[–0.154, 0.057]	.364	–0.050 (0.054)	[–0.155, 0.056]	.357
Cluster 3 (vs. Cluster 2)	–0.035 (0.047)	[–0.128, 0.057]	.456	–0.036 (0.047)	[–0.128, 0.057]	.449
Education	–0.004 (0.014)	[–0.032, 0.024]	.777	–0.005 (0.014)	[–0.032, 0.023]	.742
Age (centered)	0.015 (0.001)	[0.012, 0.017]	<.001	0.015 (0.001)	[0.012, 0.017]	<.001
Female (vs. male)	0.110 (0.038)	[0.034, 0.185]	.004	0.109 (0.038)	[0.034, 0.184]	.004
Survey year	Included	—	—	Included	—	—
Within-person health × Cluster 1	–0.036 (0.042)	[–0.118, 0.047]	.395	–0.039 (0.045)	[–0.127, 0.049]	.386
Within-person health × Cluster 3	–0.009 (0.038)	[–0.083, 0.065]	.813	–0.007 (0.040)	[–0.085, 0.072]	.869

Panel B. Random effects and model fit						
**Statistic**	**Random intercept model**			**Random slope model**		
Random intercept variance	0.923			0.929		
Random intercept SD	0.961			0.964		
Random slope variance	—			0.061		
Random slope SD	—			0.246		
Intercept–slope correlation	—			–0.140		
Residual variance	1.290			1.267		
Residual SD	1.140			1.125		
Individuals	3,293			3,293		
Person-year observations	17,102			17,102		
AIC	57,925			57,904		
BIC	58,073			58,067		
Log likelihood	–28,944			–28,931		

Likelihood-ratio test (RI vs. RS): Δχ^2^(2) = 25.54, *p* <.001

*Note.* Estimates are from maximum-likelihood random-effects within-between (REWB) models predicting life satisfaction. Cluster 2 served as the reference category. The within-person health effect represents deviations from each respondent's own average lagged health, whereas the between-person effect represents respondents' average lagged health across the observation period. Both models adjusted for education, centered age, sex, and survey-year fixed effects. The random-slope model additionally allowed the within-person health effect to vary across individuals

**Fig. 2:**
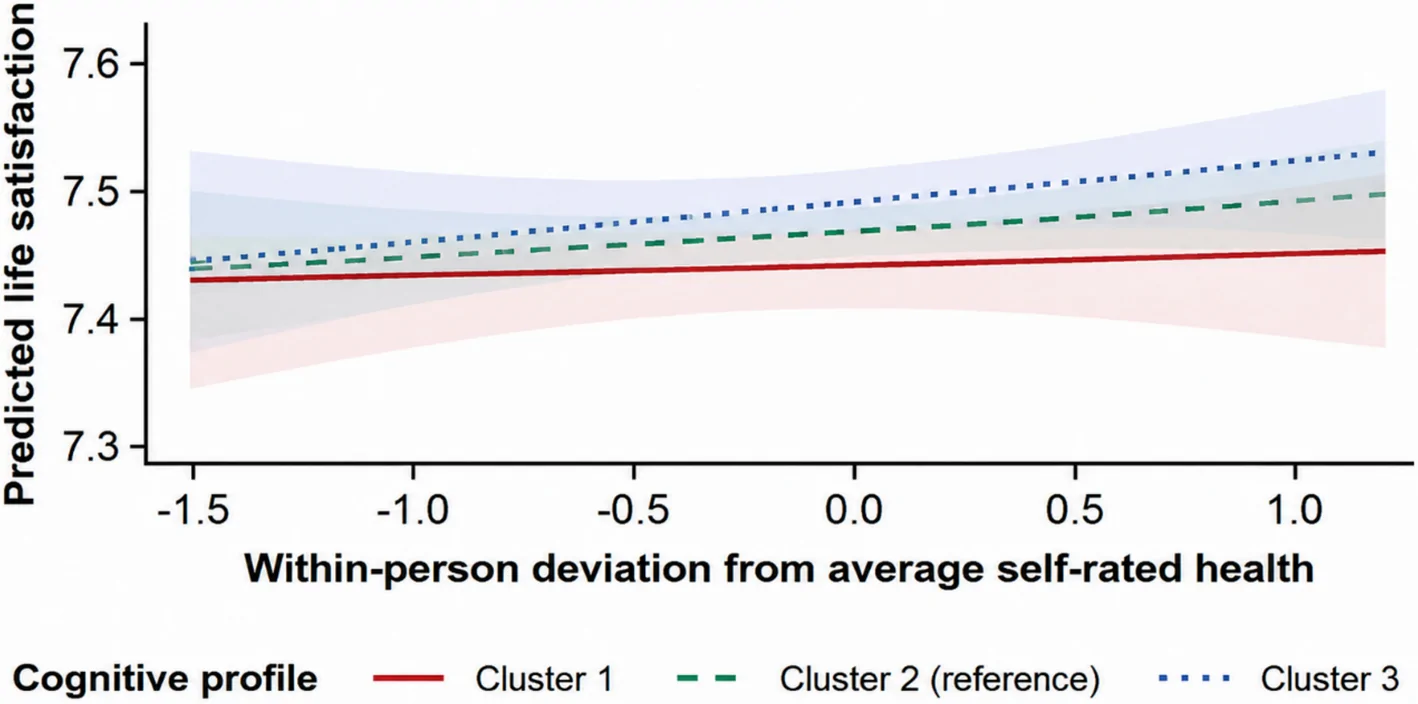
Estimated Within-Person Association Between Self-Rated Health and Life Satisfaction by Cluster *Note.* Lines show estimated marginal predictions from the random-slope REWB model; shaded areas represent 95% confidence intervals. Higher values indicate better self-rated health relative to the respondent's own average (within-person health deviation). Predictions are adjusted for model covariates. Cluster 1 was characterized by lower verbal knowledge and approximately average response-time characteristics; Cluster 2 (reference) by higher verbal knowledge and faster response times; and Cluster 3 by higher verbal knowledge, slower response times, and lower response-time variability

Figure 2 displays the estimated within-person association between self-rated health and life satisfaction for each cognitive profile. Profile-specific simple slopes were positive but varied in precision (Profile 1: *B* = 0.015, 95% CI [− 0.062, 0.092]; Profile 2: *B* = 0.054, 95% CI [0.011, 0.097]; Profile 3: *B* = 0.047, 95% CI [− 0.019, 0.113]). None of the pairwise differences between slopes was statistically significant after Bonferroni adjustment (all adjusted *p*s = 1.00; Supplementary Table S7). Although the estimated slopes differed slightly in magnitude, their 95% confidence intervals overlapped substantially. This pattern is consistent with the nonsignificant interaction terms and indicates little evidence that the within-person association between self-rated health and life satisfaction differs systematically across cognitive profiles.

Additional adjustment for positive and negative affect showed that the within-person health effect remained similar in magnitude (*B* = 0.052, *SE* = 0.021, 95% CI [0.012, 0.092]), and there remained no evidence that this association differed by cognitive profile (Profile 1 interaction: *B* = − 0.043, *SE* = 0.042, 95% CI [− 0.125, 0.040]; Profile 3 interaction: *B* = − 0.021, *SE* = 0.038, 95% CI [− 0.094, 0.053]).

In the survey-weighted sensitivity analysis, the within-person health estimate remained positive but was attenuated, and its confidence interval included zero (*B* = 0.035, *SE* = 0.025, 95% CI [− 0.014, 0.083]). The moderation results remained unchanged, with no evidence of differential within-person health effects across cognitive profiles (Supplementary Table S6).

### Exploratory between-person associations with self-rated health and subjective well-being

Exploratory between-person regression analyses examined whether cognitive cluster membership was associated with self-rated health, life satisfaction, and affect in 2014, adjusting for education, age, and sex. Compared with participants in Cluster 2, participants in Cluster 1 reported lower life satisfaction (*B* = − 0.205, *SE* = 0.071, 95% CI [− 0.344, − 0.067], *p* = 0.004), poorer self-rated health (*B* = − 0.196, *SE* = 0.039, 95% CI [− 0.272, − 0.119], *p* < 0.001), higher negative affect (*B* = 0.075, *SE* = 0.031, 95% CI [0.014, 0.137], *p* = 0.016), and lower positive affect (*B* = − 0.155, *SE* = 0.035, 95% CI [− 0.224, − 0.087], *p* < 0.001). Participants in Cluster 3 did not differ significantly from Cluster 2 in life satisfaction, self-rated health, or negative affect but reported lower positive affect (*B* = − 0.068, *SE* = 0.032, 95% CI [− 0.131, − 0.005], *p* = 0.034). Full regression results are presented in Supplementary Table S5.

## Discussion

Membership in cognitive clusters derived from verbal knowledge and response-time characteristics was associated with stable between-person differences in self-rated health, life satisfaction, and affect. Participants characterized by lower verbal knowledge reported lower life satisfaction, poorer self-rated health, higher negative affect, and lower positive affect than those in the reference cluster. Verbal knowledge is often interpreted as a marker of crystallized cognitive ability and cognitive reserve [21, 22]. These findings are broadly consistent with previous evidence linking higher cognitive functioning to better health and quality of life [16, 17].

With respect to RQ1, the longitudinal analyses provided evidence of a small positive within-person association between self-rated health and life satisfaction: when individuals reported better health than usual, they subsequently tended to report slightly higher life satisfaction. However, this association was somewhat sensitive to the analytic specification. It remained similar after additional adjustment for positive and negative affect but was weaker in the survey-weighted analysis. The average within-person association between perceived health and life satisfaction should therefore be interpreted as modest rather than as a uniformly robust effect.

With respect to RQ2, individuals differed substantially in the extent to which changes in self-rated health were associated with changes in life satisfaction, but cognitive cluster membership did not systematically account for this heterogeneity. This conclusion was consistent across the primary, affect-adjusted, and survey-weighted analyses. Cognitive reserve and vulnerability–stress perspectives suggest that greater cognitive resources may facilitate adaptation to changing health conditions through coping, compensation, and appraisal processes [23], . Based on these perspectives, we expected individuals with greater verbal knowledge and more efficient processing to show less sensitivity of life satisfaction to changes in health. The present findings did not support this expectation. The cognitive indicators examined here—verbal knowledge, processing speed, and response-time variability—were not systematically associated with individual differences in the within-person health–life satisfaction relationship.

Life satisfaction reflects a broad evaluation of one's overall life circumstances [9], and individual differences in its responsiveness to health changes may therefore depend on resources and circumstances not captured by the cognitive measures examined here. Personality, social support, coping strategies, and environmental circumstances may represent more relevant sources of heterogeneity in health-related well-being dynamics. Future research could examine these factors alongside broader measures of cognitive functioning to determine which individual characteristics contribute to differences in adaptation to changing health.

Taken together, the findings suggest a distinction between levels and dynamics of subjective health and well-being. The cognitive performance characteristics examined here were associated with stable between-person differences in self-rated health and subjective well-being but did not systematically differentiate how individuals' life satisfaction responded to changes in their perceived health over time.

### Limitations

Several limitations should be acknowledged. First, although the longitudinal design and within–between modeling allowed a clear separation of within-person change from stable between-person differences, the analyses were observational and do not support causal inference. Second, self-rated health and life satisfaction were measured annually and may therefore not capture shorter-term dynamics or acute health shocks that influence well-being over shorter time horizons.

Third, cognitive performance was assessed once, in 2014, and treated as a stable baseline indicator during the subsequent observation period. Although restricting the longitudinal analyses to observations collected from 2014 onward substantially reduced the temporal mismatch between the cognitive assessment and the longitudinal outcomes, differential age-related cognitive change may nevertheless have occurred during follow-up. Consequently, the present analyses could not account for within-person changes in cognitive functioning over time. Future studies incorporating repeated objective cognitive assessments are needed to determine whether changes in cognitive functioning influence the association between changes in self-rated health and life satisfaction.

Fourth, cognitive heterogeneity was derived from the subset of performance-based measures available in the SOEP-IS, namely verbal knowledge, mean response latency, and response-time variability. These indicators capture selected aspects of cognitive performance but do not assess other important domains, including episodic memory, executive functioning, reasoning, attention, or verbal fluency. The identified clusters should therefore be interpreted as task-specific configurations of verbal knowledge and response-time characteristics rather than comprehensive profiles of global cognitive functioning. Accordingly, the observed associations should not be generalized to cognitive functioning more broadly or to cognitive domains that were not assessed in the present study. Future research incorporating broader cognitive test batteries will be needed to determine whether the present findings extend to other aspects of cognition.

Fifth, although supplementary analyses additionally adjusted for positive and negative affect, unmeasured social or contextual factors—such as changes in the work environment, social support, or life events—may also contribute to individual differences in sensitivity to health fluctuations. Finally, although cognitive performance was assessed independently using standardized performance-based tasks, self-rated health is a multidimensional evaluative construct that may partly incorporate respondents' perceptions of their own cognitive functioning [5]. Consequently, the distinction between objectively measured cognitive performance and self-rated health is unlikely to be absolute. Although the present study minimizes shared-method bias by combining objective cognitive assessments with subjective health ratings, future studies incorporating objective health indicators alongside self-rated health may further disentangle these related constructs.

## Conclusion

This study advances understanding of health-related well-being by distinguishing between stable between-person differences and dynamic within-person responses to health changes. Using longitudinal panel data and performance-based cognitive clusters, we found that cognitive performance was associated with stable differences in self-rated health, life satisfaction, and affect but did not explain why individuals differed in the extent to which changes in self-rated health translated into changes in life satisfaction over time. By distinguishing between-person differences from within-person health-related changes, the present study contributes to a more nuanced understanding of the relationships between cognition, perceived health, and subjective well-being.

## Supplementary Information

Supplementary Material 1.

## Data Availability

Data Availability Statement The data that support the findings of this study are available from the German Socio-Economic Panel (SOEP) at the German Institute for Economic Research (DIW Berlin). The study uses data from the SOEP Core (v40.1) and the SOEP Innovation Sample (SOEP-IS) cognition module (2014). These are third-party data and are not publicly available due to data protection regulations. Access to SOEP data is available to qualified researchers for scientific use upon application and approval by DIW Berlin and after signing a data use agreement. Information on how to apply for access is available at: https://www.diw.de/soep The authors confirm that they accessed the data through this standard application process and did not receive special privileges.
