# Supplementary material for: Cognitive heterogeneity, health dynamics, and subjective well-being across adulthood: evidence from the German SOEP longitudinal panel
Source: BMC Public Health. 2026 Sep 11;26:2584. doi: 10.1186/s12889-026-29407-5 (PMC13563874; doi:10.1186/s12889-026-29407-5)
Supplement: Supplementary file 1 [file 12889_2026_29407_MOESM1_ESM.pdf]

Supplement

Figure S1. Elbow Plot for Selecting the Number of Cognitive Profiles (SOEP-IS 2014)

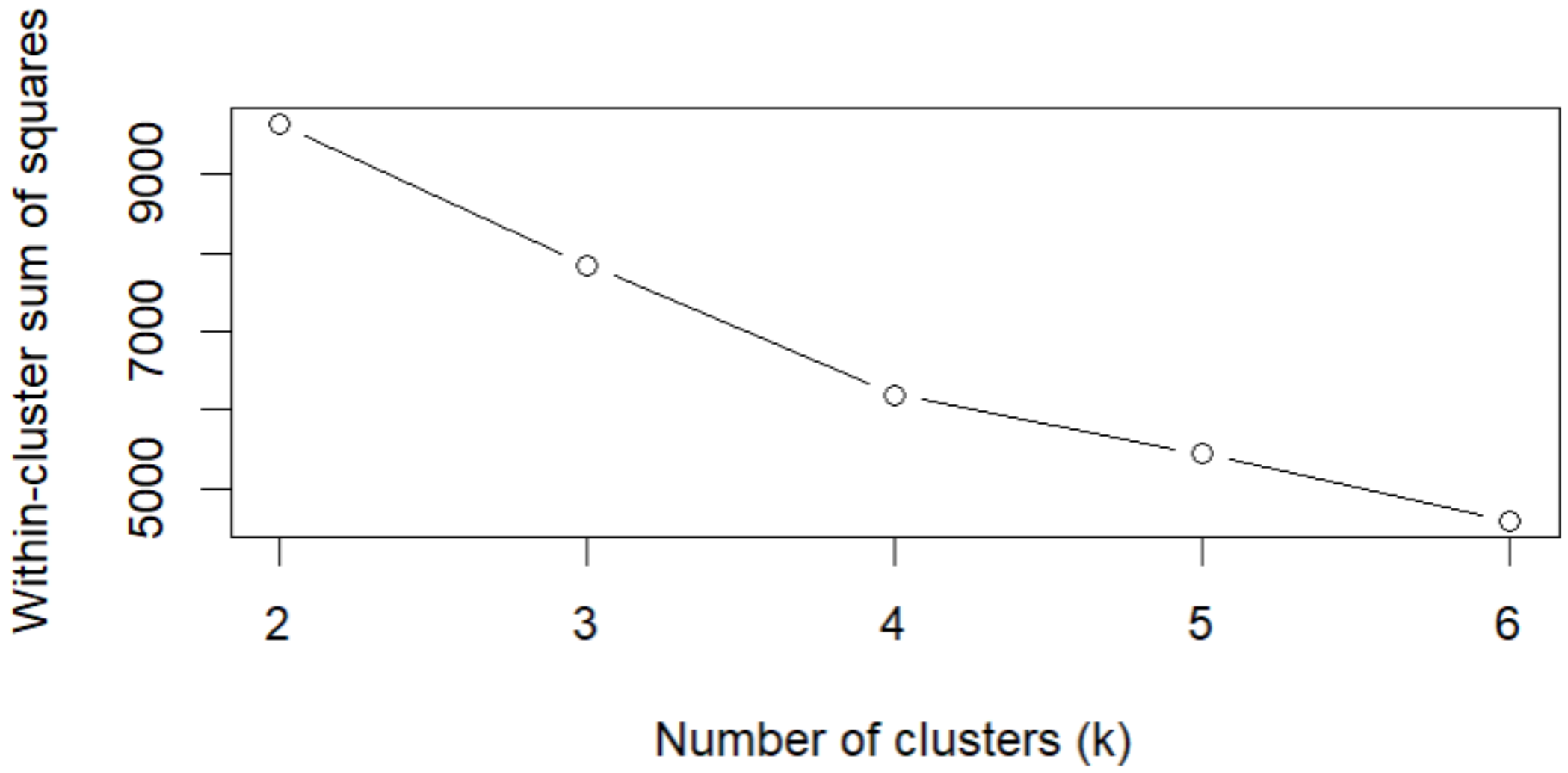

Note. The figure plots the within-cluster sum of squares (WCSS) for k-means solutions with 2 to 6 clusters based on standardized word test performance, log-transformed processing speed, and processing speed variability. The reduction in WCSS is steep up to three clusters and levels off thereafter, indicating diminishing improvements in within-cluster homogeneity for additional clusters. Based on this elbow pattern and the substantive interpretability of the resulting profiles, a three-cluster solution was selected for the main analyses.

Table S2 *Missing Data in the 2014 Cognitive-Profile Sample*

**Variable**	**Total *N***	**Nonmissing, *n***	**Missing, *n***	**Missing, %**
Verbal knowledge score	4,067	4,067	0	0.00
Mean log response latency	4,067	4,067	0	0.00
Response-time variability	4,067	4,067	0	0.00
Life satisfaction	4,067	4,067	0	0.00
Self-rated health	4,067	4,066	1	0.02
Negative affect	4,067	4,065	2	0.05
Positive affect	4,067	4,067	0	0.00
Education	4,067	4,035	32	0.79

**Variable**	**Total *N***	**Nonmissing, *n***	**Missing, *n***	**Missing, %**
Age	4,067	4,067	0	0.00
Sex	4,067	4,067	0	0.00

Note. Negative SOEP missing and filter codes were recoded as missing before scale construction and analysis. Cognitive-profile derivation required complete data on all three cognitive indicators; accordingly, these indicators have no missing values in the clustering sample.

Table S3 *Availability of Variables in the Broad Post-2014 Person-Year Panel*

**Variable**	**Person-years, *N***	**Available, *n***	**Unavailable or missing, *n***	**%**
Cognitive profile available	54,433	24,825	29,608	54.40
Life satisfaction	54,433	47,808	6,625	12.17
Self-rated health	54,433	49,053	5,380	9.88
Education	54,433	47,883	6,550	12.03
Age	54,433	54,433	0	0.00
Sex	54,433	54,421	12	0.02

Note. Unavailability of a cognitive profile reflects nonparticipation in the 2014 SOEP-IS cognition module and should not be interpreted as item-level missingness.

Table S4 *Baseline Characteristics of Respondents Included in and Excluded From the Final Life-Satisfaction Model*

**Characteristic**	Included (*n* = 3,293)	Excluded (*n* = 774)	SMD	*p*
**Age, years, *M* (*SD*)**	51.37 (17.57)	47.98 (19.74)	0.19	< .001
**Education, *M* (*SD*)**	2.50 (1.42)	2.54 (1.50)	−0.02	.597
**Life satisfaction, *M* (*SD*)**	7.47 (1.62)	7.39 (1.79)	0.05	.226
**Self-rated health, *M* (*SD*)**	3.42 (0.94)	3.40 (1.03)	0.02	.617
**Negative affect, *M* (*SD*)**	2.24 (0.76)	2.24 (0.75)	0.00	.986
**Positive affect, *M* (*SD*)**	3.62 (0.82)	3.62 (0.84)	0.01	.773
**Verbal-knowledge score, *M* (*SD*)**	29.92 (3.65)	28.91 (4.24)	0.27	< .001
**Mean log response latency, *M* (*SD*)**	3.89 (0.08)	3.89 (0.08)	0.01	.840
**Response-time variability, *M* (*SD*)**	26.16 (2.64)	26.14 (1.94)	0.00	.889
**Female, *n* (%)**	1,749 (53.1%)	364 (47.0%)	—	.003
**Cognitive profile, *n* (%)**				< .001
**Cluster 1**	558 (16.9%)	198 (25.6%)	—	
**Cluster 2**	1,902 (57.8%)	387 (50.0%)	—	
**Cluster 3**	833 (25.3%)	189 (24.4%)	—	

Note. Included respondents contributed to the final complete-case life-satisfaction model; excluded respondents belonged to the 2014 cognitive-profile sample but did not meet the final longitudinal model requirements. SMD = standardized mean difference. Positive SMD values indicate higher means in the included group. Self-rated health was reverse-coded so that higher scores indicate better health. Continuous-variable *p*-values are based on independent-samples Welch *t* tests. The *p*-value for sex is based on a chi-square test. The overall distribution of cognitive profiles differed between included and excluded respondents, $\chi^2(2) = 32.04$, $p < .001$.

Table S5

Exploratory Between-Person Regression Models Predicting Self-Rated Health and Subjective Well-Being in 2014

**Outcome**	**Comparison**	***B***	**SE**	**95% CI**	***p***	***N***
Life satisfaction	Cluster 1 vs. Cluster 2	-0.205	0.071	[-0.344, -0.067]	.004	4,035
	Cluster 3 vs. Cluster 2	-0.103	0.065	[-0.231, 0.024]	.112	4,035
Self-rated health	Cluster 1 vs. Cluster 2	-0.196	0.039	[-0.272, -0.119]	< .001	4,034
	Cluster 3 vs. Cluster 2	-0.062	0.036	[-0.132, 0.008]	.084	4,034
Negative affect	Cluster 1 vs. Cluster 2	0.075	0.031	[0.014, 0.137]	.016	4,033
	Cluster 3 vs. Cluster 2	-0.006	0.029	[-0.063, 0.051]	.835	4,033
Positive affect	Cluster 1 vs. Cluster 2	-0.155	0.035	[-0.224, -0.087]	< .001	4,035
	Cluster 3 vs. Cluster 2	-0.068	0.032	[-0.131, -0.005]	.034	4,035

Note. Estimates are from separate ordinary least squares regression models using the 2014 SOEP-IS cognition sample. Cluster 2 served as the reference category. All models adjusted for educational attainment, age in 2014, and sex. Higher self-rated health scores indicate better health. Positive coefficients indicate higher outcome values relative to Cluster 2, whereas negative coefficients indicate lower values. Sample sizes vary slightly because of outcome-specific missing data.

Table S6 Sensitivity Analyses for the Within-Person Association Between Self-Rated Health and Life Satisfaction

**Effect**	Primary model, *B* (*SE*), 95% CI	Affect-adjusted model, *B* (*SE*), 95% CI	Survey-weighted model, *B* (*SE*), 95% CI
**Within-person health**	0.054 (0.022), [0.011, 0.096]	0.052 (0.021), [0.012, 0.092]	0.035 (0.025), [−0.014, 0.083]

**Effect**	Primary model, *B* (*SE*), 95% CI	Affect-adjusted model, *B* (*SE*), 95% CI	Survey-weighted model, *B* (*SE*), 95% CI
**Within-person health × Profile 1**	−0.039 (0.045), [−0.127, 0.049]	−0.043 (0.042), [−0.125, 0.040]	0.015 (0.048), [−0.080, 0.110]
**Within-person health × Profile 3**	−0.007 (0.040), [−0.085, 0.072]	−0.021 (0.038), [−0.094, 0.053]	0.021 (0.045), [−0.068, 0.110]
**Individuals, *n***	3,293	3,293	3,293
**Person-year observations, *n***	17,102	17,092	17,091

Note. Values are unstandardized regression coefficients (*B*), with standard errors in parentheses and 95% confidence intervals in brackets. All models are random-slope random-effects within-between (REWB) models predicting life satisfaction. Models include the within-person component of lagged self-rated health, between-person mean lagged self-rated health, cognitive profile, education, centered age, sex, survey-year fixed effects, and interactions between within-person health and cognitive profile. Cognitive Profile 2 served as the reference category. The affect-adjusted model additionally includes positive and negative affect. The survey-weighted model applies SOEP cross-sectional person weights (*phrf*) at the person-year level; weights were standardized to a mean of one and trimmed at the 1st and 99th percentiles.

Table S7 Cognitive-Profile-Specific Within-Person Associations Between Self-Rated Health and Life Satisfaction

Panel A. Profile-Specific Simple Slopes

**Cognitive profile**	***B***	***SE***	**95% CI**	***p***
Profile 1	0.015	0.039	[−0.062, 0.092]	.707
Profile 2 (reference)	0.054	0.022	[0.011, 0.097]	.014
Profile 3	0.047	0.034	[−0.019, 0.113]	.160

Panel B. Pairwise Contrasts of Profile-Specific Slopes

**Comparison**	**Difference in *B***	***SE***	**95% CI**	**Bonferroni-adjusted *p***
Profile 2 – Profile 1	0.039	0.045	[−0.069, 0.146]	1.000

Comparison	Difference in *B*	*SE*	95% CI	Bonferroni-adjusted *p*
Profile 2 – Profile 3	0.007	0.040	[−0.089, 0.102]	1.000
Profile 1 – Profile 3	−0.032	0.052	[−0.156, 0.091]	1.000

Note. Profile-specific slopes represent estimated within-person associations between lagged self-rated health and life satisfaction from the primary random-slope random-effects within-between (REWB) model. Higher values of within-person health indicate better self-rated health relative to the respondent's own average. The model adjusted for between-person mean lagged self-rated health, cognitive profile, education, centered age, sex, and survey-year fixed effects. Cognitive Profile 2 served as the reference category in the primary model. Confidence intervals and tests are based on asymptotic inference. Pairwise contrasts in Panel B use Bonferroni-adjusted confidence intervals and *p*-values.

Table S8 Fit and Classification Statistics for Supplementary Latent Profile Models

Requested profiles	BIC	ICL	Smallest profile, *n* (%)	Largest profile, *n* (%)	Occupied profiles
2	−34,696.88	−39,345.85	1,385 (34.05)	2,682 (65.95)	2
3	−33,757.11	−37,958.81	256 (6.29)	2,858 (70.27)	3
4	−33,417.61	−37,742.65	100 (2.46)	2,283 (56.13)	4
5	−33,450.73	−38,862.45	100 (2.46)	1,837 (45.17)	4
6	−30,853.64	−34,153.02	5 (0.12)	3,169 (77.92)	5

*Note***.** Latent profile models were estimated using standardized verbal knowledge, log-transformed mean response latency, and response-time variability, assuming equal within-profile variances and zero within-profile covariances (EEI specification). BIC = Bayesian information criterion; ICL = integrated classification likelihood. Higher (less negative) BIC and ICL values indicate better fit. The requested five- and six-profile solutions yielded fewer populated profiles than specified and were therefore not interpreted substantively.
